# Clinical Features, Pathophysiology, and Treatment of Levodopa-Induced Dyskinesias in Parkinson's Disease

**DOI:** 10.1155/2012/943159

**Published:** 2012-10-17

**Authors:** J. Guridi, R. González-Redondo, J. A. Obeso

**Affiliations:** Department of Neurosurgery and Neurology, Clinica Universidad de Navarra, 31008 Pamplona, Spain

## Abstract

Dyskinetic disorders are characterized by excess of motor activity that may interfere with normal movement control. In patients with Parkinson's disease, the chronic levodopa treatment induces dyskinetic movements known as levodopa-induced dyskinesias (LID). This paper analyzed the pathophysiology, clinical manifestations, pharmacological treatments, and surgical procedures to treat hyperkinetic disorders. Surgery is currently the only treatment available for Parkinson's disease that may improve both parkinsonian motor syndrome and LID. However, this paper shows the different mechanisms involved are not well understood.

## 1. Introduction

Hyperkinetic or dyskinetic disorders are characterized by excessive muscular activity that may interfere with normal movement control. Dyskinesias include different types of movement disorders such as chorea-ballism, dystonia, myoclonus, tics, and tremor. In patients with Parkinson's disease (PD), chronic levodopa treatment may induce various dyskinetic movements (levodopa induced dyskinesias (LID)) which are classified according to the phenomenology and also their temporal presentation in relation with the effect of levodopa.

The association between levodopa and the induction of dyskinesias was recognized soon after the introduction of levodopa [1, 2]. In the past, levodopa therapy was associated with the development of motor complications in about 80% of patients within 5 years of treatment [3, 4]. In patients with young onset PD, the incidence of LID was higher and ensued more rapidly [2, 5]. Currently, with the introduction and widespread use of dopaminergic agonists, the overall treatment exposure to levodopa is decreasing, especially in the first years of treatment; nevertheless, progression of the nigrostriatal deficit will facilitate the onset of LID at a later point in time. Thus, LID continues to be a common and important cause of disability in PD and one of the main reasons for recommending surgical treatment. 

In this paper we describe the major clinical features, main pathophysiological and pharmacological abnormalities associated with LIDs, and the drug and surgical treatments currently available.

## 2. Clinical Presentation

**Figure 1:**
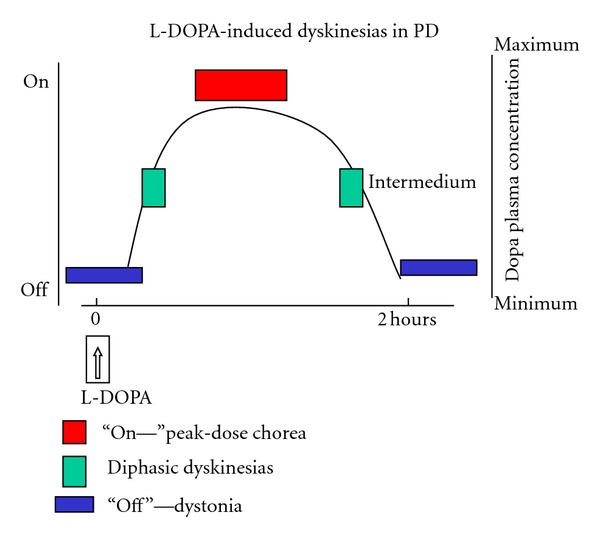
Relationship between LID and DOPA plasma level. “Peak of dose” or “on” period dyskinesia is correlated to high level of levodopa and in parallel with the maximal clinical benefit. Diphasic dyskinesia appears at the onset and offset of the levodopa effect in relationship with increment or decrement of plasma level. “Off” period dystonia is characterized by painful postures in lower extremities and is correlated with the lowest levodopa level. Generally a full spectrum of the three types is present in patients with motor fluctuations.

LID may be divided into various presentation forms (Figure 1) [6].

1. “Peak dose” or “on” period dyskinesia related to high plasma levels of levodopa, in parallel with the maximal antiparkinsonian benefit. These are typically choreic in nature and predominantly involve the neck, trunk, and upper limbs, but dystonic movements may also occur.
2. Diphasic dyskinesia appears at the onset and offset of the levodopa effect, coinciding with arising and decaying plasma levodopa levels. This is characterized by repetitive and stereotyped repetitive, slow (<4 Hz) movements of the lower limbs often coinciding with 4 Hz tremor in the upper limbs [4], indicating the patient is not fully “on”. In severe cases, the movements of the legs may lose the repetitive and stereotypic nature and resemble ballism. In a small proportion of patients, diphasic dyskinesias are very prominent while walking, drastically interfering with gait, and giving rise to a picturesque pattern [7]. Dystonic posture may also occur, although much less frequently.
3. “Off” period dystonia, characterized by fixed and painful postures more frequently affecting the feet, but which can be segmental or generalized in distribution.

A combination of any of these 3 types or indeed, all of them, may be observed in some patients throughout the levodopa (“off-on-off”) cycle. Until now LID, by definition, were associated with levodopa intake and, to a much lesser extent, with dopamine agonists used in monotherapy. Two more recent situations whereby dyskinesias can be induced in patients with PD despite not being treated with dopaminergic drugs are (1) patients treated with fetal cell transplants [8]; (2) patients treated with deep brain stimulation (DBS) of the subthalamic nucleus (STN) [9]. The former has no practical implications as experimental trials with fetal cell transplants are not a therapeutic option, but STN-DBS is fairly frequently applied. In the latter instance, adjusting the current parameters usually results in control of dyskinesias.

## 3. Pathophysiology of LID

Levodopa is converted into dopamine (DA) in many brain regions and “a priori” there are several sites where its dyskinesogenic effect could occur. The striatal origin of LIDs was suspected as soon as the problem was recognized in the early 1970's but there were no experimental or clinical proofs. Direct proofs arose unexpectedly when fetal mesencephalic cells transplanted into the striatum in experimental trials for PD were associated with dyskinesias with a similar clinical pattern than LID [10, 11]. 

**Figure 2:**
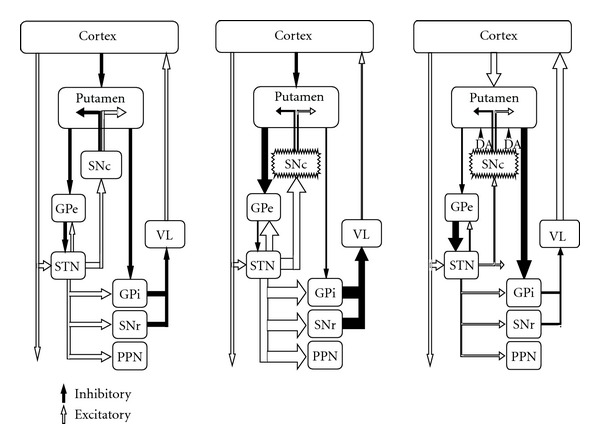
Classic model of basal ganglia in normal condition, parkinsonian, and dyskinetic conditions. During LID the different population of striatal cells from direct and indirect circuit are opposite to parkinsonian state. LID would result from a decrease in the inhibitory pathway by striatal neurons in the indirect pathway to the GPe, leading to an inhibitory increment over the STN and consequently reducing STN and GPi/SNr activity. This is facilitated by the increase in the inhibitory striatal activity of GPi by the direct pathway from striatum.

Two main factors are involved in the origin of LID. (1) Degree of dopaminergic nigro-striatal depletion, which is related to disease duration and severity. (2) The pharmacokinetics and mechanism of action of levodopa, which delivers a discontinuous or pulsatile stimulation of dopaminergic receptors [3, 12]. Together, degree of nigro-striatal lesion and the action of levodopa interact to induce changes in corticostriatal transmission and plastic synaptic abnormalities in striatal spiny neurons, which ultimately may alter the physiological activity of striatopallidal circuits, leading to abnormal pattern of neuronal activity underlying LID [13, 14]. A direct demonstration of the link between short acting dopaminergic stimulation and changes in basal ganglia output was provided several years ago. It was shown that once or twice a day levodopa or apomorphine administration in parkinsonian monkeys induced dyskinesias which were associated with a reduction in the main firing frequency of globus pallidus internus (GPi) neurons [15, 16]. Similar results have been described in parkinsonian patients who were administered apomorphine during pallidal surgery. Here, the turning from the “off” parkinsonian condition to the “on” mobile state plus LID was associated with a significant reduction in the mean neuronal firing rate of the GPi and STN [17–19]. In addition, STN and GPi activity was decreased when assessed by regional brain uptake of 2-deoxyglucose, which measures afferent synaptic activity, in MPTP monkeys with dyskinesias induced by dopaminergic drugs [20]. Thus, reduced GPi inhibitory output activity to the thalamus leads to disinhibition of the thalamo-cortical projection, facilitating the abnormal recruitment of cortical motor areas which ultimately give rise to dyskinetic movements. In simple terms therefore, dyskinesias in general and LID in particular may be understood as the reverse of the parkinsonian state, whereby the latter is mainly characterized by overactivity of the STN and GPi output, leading to over-inhibition of the thalamus and decreased thalamocortical activity (Figure 2) [20–24].

The metabolic activity reduction and firing reduction and firing frequency changes in firing pattern of GPi activity to the thalamus are thought to produce an increase in thalamocortical drive leading to dyskinesia.

Which striatopallidal circuits, if any, may be preferentially mediate LID has been a matter of discussion over the years. D_2_ mediated activation of the striato-pallidal projections in the “indirect” basal ganglia circuit was favored for a long time. Thus, pharmacological manipulation of the “indirect” circuit induces dyskinesias in monkeys which are similar to LID. For example, this is achieved by injecting bicuculline, a *γ*-amino butyric acid (GABA) antagonist into the globus pallidus externus (GPe), which results in increased GPe efferent activity and overinhibition of the STN [25] or by blocking STN glutamatergic projection, which provokes GPi neuronal hypoactivity and involuntary movements in the monkey [26, 27]. Moreover, it is well known that STN lesion induces hemichorea-ballism, and both deep brain stimulation (DBS) and subthalamotomy in PD patients may induce dyskinesias that are identical to those triggered by levodopa. On the other hand, more recently molecular changes in the striatum and the effects of some dopaminergic drugs have suggested, that LID are mediated by D_1_ receptor activation in the “direct” circuit [28, 29]. Thus, increased activity in the signaling by activation of D_1_ receptors has been encountered both in animal models and PD patients with LID [30–33]. D_1_ receptor is abnormally abundant at the plasma membrane of striatal neurons and it seems to be dysregulated in LID by alterations in intraneuronal trafficking [34]. In addition, some interesting findings have suggested a relevant role for D_3_ receptor in the pathophysiology of LID [35, 36].

It is also important to consider the changes related to glutamatergic striatal input. The striatum receives massive cortical and thalamic glutamatergic inputs, which are increased in the parkinsonian state [37]. This has been suggested as the mechanism mediating loss of spines in medium spiny neurons [38], which in turn could render the striatum vulnerable to large changes in dopamine availability following levodopa treatment in PD. Recent evidence suggests that the expression, proportion and location of striatal NMDA glutamate receptors may play a paramount role in the molecular mechanisms mediating LID. In the 6-hydroxydopamine (OHDA) rat model it has been shown that the ratio of NR_2b_/NR_2a_ is increased and there is a shift to the extra-synaptic space of the NR_2b_ receptor subunit in dyskinetic rats [39].

Recently, optogenetics was applied to selectively block the protein DARPP-32 in medium spiny neurons of the “direct” striatonigral projection, resulting in marked LID reduction in the rat model, whereas blockade of striatopallidal neurons giving rise to the “indirect circuit” produced a robust increase in locomotors activity and reduced cataleptic response to haloperidol [40].

Finally, dopaminergic drugs act not only in the striatum but also on other basal ganglia nuclei, the thalamus and cortex, all of which are dopamine depleted in variable extent in PD. The possible action of levodopa and other dopaminergic drugs modulating firing activity of the GPe, GPi and STN should not be underestimated and is still pending definitive studies.

Altogether, there is increasing evidence that overlapping mechanisms underlie the appearance of LID. They seem to converge in alterations of the striatal synaptic function in response to the loss of dopaminergic input and to subsequent replacement of dopamine by pharmacological means [29]. This concept, defined as striatal plasticity, occurs through functional processes such as long term potentiation, long term depression, or a maladaptive form of plasticity invoked as depotentiation [33, 41]. In the presence of exogenous levodopa, distinct patterns of synaptic aberrant plasticity developed in both the direct and indirect pathways, and so a new perspective is open whereby LID in PD could be considered as a network disorder [42]. Indeed, two recent studies comparing LID versus non-LID groups of patients found an increase in the structural signal of the gray-matter focused on the inferior frontal gyrus (IFG) particularly in the right hemisphere, whereas a functional MRI study pointed to an increased task-related activity in the supplementary motor area and reduced activity in the right IFG. These data suggest that changes in the right IFG reflect neuroplasticity following from years of increased use of executive control to override involuntary movements in LID [43].

In conclusion, the dopaminergic system controls the excitability of the striatum and other basal ganglia nuclei leading to modulation of neuronal firing rates and patterns. LID may originate in striatal spiny neurons, mainly in the putamen leading to reduced mean discharge rate, abnormal firing pattern, and pathological oscillatory activity that are transmitted throughout striatopallidal projection to the thalamocortical projection.

## 4. LID Pharmacological Treatments

Three main therapeutic strategies have been used to treat LID in PD.

1. Prevention of LID development by early use of dopamine agonist drugs and reduced levodopa dose intake at the beginning of treatment.
2. Symptomatic treatment, once LID developed, with putative antidyskinetic interventions.
3. Reverting dyskinesias by continuous dopaminergic stimulation to achieve a wider therapeutic window, reducing “off” hours while improving dyskinesias.

### 4.1. Prevention of LID

The use of neuroprotective drugs to slow disease progression has been extensively explored. L-deprenyl(selegiline), in an extension of the DATATOP study, failed to produce a significant reduction in the incidence of dyskinesias [44]. 

The only group that has demonstrated to some extent a reduction in the risk of developing dyskinesias is the dopamine agonists. Several placebo-controlled studies compared the evolution of patients initiated with a dopamine agonist (ropinirol, pramipexol, and cabergoline) and standard levodopa. Rascol et al. in a comprehensive, double-blind parallel study, compared the efficacy of ropinirol and levodopa over a period of 5 years in 268 patients with early PD [5]. The analysis of the time to onset of dyskinesia showed a significant difference in favor of ropinirol. The cumulative incidence of dyskinesia at fifth year, regardless of levodopa supplementation, was 20% in the ropinirol group and 45% in the levodopa group. The mean daily dose of ropinirol was 15 mg but the majority of the patients enrolled in that group required supplementary treatment with levodopa [5]. When patients receiving ropinirole monotherapy required the addition of levodopa, the risk of developing dyskinesias increased, and eventually, during followup, did not differ significantly from that associated with levodopa alone [45]. The use of ropinirole as monotherapy with only later addition of levodopa over 10-year follow-up delayed the onset of dyskinesias by up to 3 years [46]. Moreover, the prolonged-release form of ropinirole recently demonstrated a delay in the onset of dyskinesias compared with increasing doses of levodopa [47]. These clinical observations under control conditions confirmed experimental data in the MPTP monkey showing that ropinirol alone or in combination with low-dose levodopa delayed dyskinesia onset while improving motor performance [48].

The CALM-PD was a randomized controlled trial that evaluated the risk of developing dyskinesias in patients with early PD treated initially with either pramipexole or levodopa, followed by a maintenance phase during which open-label levodopa-carbidopa was permitted as needed [49]. After 24 months, pramipexole-treated patients were receiving a mean daily dose of 2.78 mg pramipexole plus 264 mg levodopa, compared with 509 mg levodopa for those receiving only this agent. There were fewer pramipexole-treated patients that reached the primary endpoint of time to first occurrence of wearing off, dyskinesias, or on-off motor fluctuations (27.8% versus 50.7%). Patients in the pramipexole group also had a significantly lower incidence of dyskinesias (9.9% versus 30.7%) [49]. After a mean 6-year follow-up, over 90% of patients ended up receiving levodopa therapy regardless of their initial treatment assignment. Compared to those taking pramipexole, patients initially treated with levodopa had significantly more dyskinesias (20.4% versus 36.8%), but there was no difference in the incidence of disabling or painful dyskinesias [50].

The ergot derivativecabergoline holdsa long half-life (*≈*72 hours) and therefore may be administered once daily. In a double-blind multicenter trial on 419 patients naive to treatment, comparing cabergoline and levodopa as initial therapy for PD, motor complications were significantly delayed and occurred less frequently in cabergoline-treated patients compared to levodopa-treated patients [51]. 

An evidence-based review compared the results of studies published on early treatment of PD with dopamine agonists with similar studies using levodopa [52]. Cabergoline, pramipexole, and ropinirol were similarly effective in reducing the risk of LID, although reduction was slightly greater for pramipexole and ropinirol than for cabergoline. The latter is no longer used widely because of the associated risk of cardiac valvulopathy [53].

A concern encountered in the three studies was that, whereas treatment with a dopamine agonist reduced the risk of dyskinesia, this was associated with less antiparkinsonian benefit. Currently, three dopamine agonists provide longer stimulation of DA receptors, by delay-release per oral route (for pramipexol and ropinirole) and transdermal application (rotigotine). The efficacy of these new dopamine agonists formulations on LID has not been specifically assessed yet. It remains also open to future analysis to determine whether the initial benefit on LID of treatment with a dopamine agonist is carried forward over the long-term evolution once levodopa is added to the regimen. In addition, several issues related to the design of the studies have been raised by critical voices. Our own view, which is generally shared by most movement disorder neurologists, is that the severity of LID observed in clinical practice has been considerably reduced over the last decade, coinciding with the earlier use of dopamine agonists and the associated possibility of reducing levodopa daily dose. Thus, while more definitive data are being compiled, we favor the prevailing concept of starting therapy with a dopamine agonist, particularly in patients who are 65 years old or younger at the time of diagnosis. This approach has been tempered by the more recent realization of a variety of impulse control disorders (ICD) associated with the use of dopamine agonists. Whether or not pathological impulsivity in PD patients will be also reduced by the use of long-acting dopamine agonists, it is too early to tell. We hope this will be the case by the analogy and shared pathophysiological mechanisms of LID and ICD [54].

### 4.2. LID as a Clinical Management Problem: Symptomatic Treatments

This is the commonest clinical scenario. Patients have already developed LID and the clinician has to attempt to control the abnormal movements by adjusting antiparkinsonian drugs or adding agents capable of reducing LID without increasing motor disability. The difficulty in achieving therapeutic efficacy is directly related to the severity and complexity of PD in each individual subject. Thus, LID are relatively easy to control when they are mild and occur in patients with a wide therapeutic window, but may be difficult or impossible to treat pharmacologically in advanced patients who exhibit all forms of LID and fall into severe “off” episodes when they are not dyskinetic. We shall review here the different individual pharmacological approaches available to treat LID but commonly, in many instances of clinical practice one needs to combine several options aiming to control both fluctuations and dyskinesias.

#### 4.2.1. Dopamine Agonists

Any one of the above mentioned dopamine agonists may be added with the intention of reducing levodopa dose and avoiding peak of dose on-dyskinesias associated with high levodopa plasma levels while controlling the severity of “off” motor state. Belanger et al. first examined the possibility of reducing LID by using a small dose of cabergoline [55]. During treatment, they found LID in the levodopa group but not in the levodopa + cabergoline group, which suggests that a small dose of a long-acting D_2_ agonist combined with low doses of levodopa could reduce the incidence of LID in patients with PD. This study supports a commonly applied clinical strategy. The practical problem in many instances arises when the reduction in levodopa doses precludes achieving a sufficiently good anti-parkinsonian response, a situation poorly tolerated by most patients.

A partial D_2_ receptor agonist may represent an interesting alternative for the treatment of PD and dyskinesias. These drugs, characterized by having lower intrinsic activity at the receptor level than full agonists, act as either functional agonist or antagonist, depending on the levels of endogenous dopamine. Preclamol has a selective dopamine mixed agonist-antagonist profile for both pre and postsynaptic receptors. Its action in patients with disabling “on-off” fluctuations was compared against placebo and subcutaneous apomorphine [56], showing a mild but significant anti-akinetic effect which was of lesser magnitude than that achieved with subcutaneous apomorphine but caused less dyskinesia. Aripiprazole is an antipsychotic drug showing partial agonist activity for D_2_ and 5HT_2A_, and antagonist for 5HT_2A_ receptors. Lieberman postulated that this drug may be able to reduce dyskinesias without enhancing parkinsonism [57], and a small pilot study was positive, [58]. However further studies are required to investigate its antidyskinetic capacity.

#### 4.2.2. Dopamine Antagonists

The use of drugs that block the dopaminergic system has been a classical approach for the treatment of dyskinesias in general. D_2_ antagonists, like haloperidol, olanzapine, tiapride, and sulpiride, and presynaptic dopamine-depleting drugs, like reserpine and tetrabenazine, have all proven useful in the management of hemichorea-ballism, tardive dyskinesias, and tics. These same drugs are also effective in reducing or suppressing LID in PD, but this is invariably associated with marked motor worsening after a variable period (ranging from hours to weeks). In clinical practice, therefore, they are neither useful nor recommended. 

Recent observations increasingly suggest that atypical neuroleptic drugs, which are able to block D_3_ receptors preferentially, can be beneficial for patients with movement disorders. Oh et al. evaluated the effects of an atypical antipsychotic drug which is antagonistic of 5HT_2A/C_ and D_2/3_ receptors, quetiapine, on motor behavior in the OHDA lesioned rat, and in MPTP treated monkeys [59]. In unilaterally lesioned rats, quetiapine reversed the shortening of the motor response to levodopa challenge produced by treatment during 3 weeks with levodopa twice daily. Quetiapine also normalized the short-duration response to acute injection of agonists either for D_1_ receptor (SKF38392) or D_2 _(quinpirole) in rats that had received levodopa in chronic administration. Quetiapine had no effect on parkinsonian manifestations when given alone to OHDA lesioned rats or MPTP monkeys, but did substantially reduce LID when administered together with levodopa. Katzenschlager et al. assessed the effect of quetiapine on dyskinesias in a double-blind cross-over study in 9 patients with PD, receiving different doses of quetiapine or placebo at night [60]. On 50 mg/day quetiapine, a slight reduction in LID severity was observed on a visual analog scale but this improvement was not reflected in the patients' overall impression of treatment effect. Durif et al. investigated the efficacy of clozapine in the treatment of LID in 50 patients during a 10-week, double-blind, placebo-controlled, multicenter trial. During a levodopa challenge the maximal LID score was significantly decreased in the clozapine group (mean dose *≈*40 mg/day), which led to the conclusion that clozapine is effective in the treatment of LID in severe PD [61].

#### 4.2.3. Glutamatergic Antagonists

The N-methyl-D aspartate (NMDA) receptor is thought to mediate excitotoxicity in the basal ganglia, but the use of NMDA antagonists in humans has generally been limited because of adverse effects associated with a non-selective blockade. Metman et al., in a double-blind cross-over study, showed that 3 weeks' treatment with dextrometorphan was able to reduce dyskinesias by 30–40% while maintaining the response to levodopa. In recent years amantadine, which is believed to increase dopamine release from presynaptic uptake sites, has become popular as an antidyskinetic drug based on its putative anti-NMDA action [62]. Del Dotto et al. evaluated the effect of a 2-hour intravenous amantadine or placebo infusion against LID in 9 PD patients with motor fluctuations and severely disabling peak-dose dyskinesias [63]. Intravenous amantadine acutely improved LID by 50%, without losing the antiparkinsonian benefit of levodopa along the 5-week, double-blind cross-over trial. In another study, Luginger et al. assessed LID severity by self-scoring diaries after oral levodopa challenges and found them to be reduced by approximately 50% after amantadine treatment compared with baseline or placebo control [64]. Further studies also found a positive effect for amantadine on LID [65, 66]. Moreover, in a recent trial in advanced PD patients receiving amantadine continuously over 1 year, a withdrawal of amantadine led to a significant increase of dyskinesias in those patients when double-blind switched to placebo, while no change occurred in those maintained on amantadine. This supports the notion of a sustained antidyskinetic effect of amantadine beyond one year of therapy. Our own view is that, on an individual basis, amantadine may result in a drastic amelioration of LID and is therefore worth trying in the absence of contraindications. The antidyskinetic effect is probably exerted at the level of the STN as amantadine failed to control dyskinesias evoked by subthalamotomy in patients who had previously responded markedly well [67].

Merello et al. evaluated the efficacy of memantine on the pharmacological response to levodopa and the induction of LID [68]. In 12 patients, in opposition to recent findings with amantadine, no effect on LID was observed. Nevertheless, several reports described a benefit of memantine in PD patients with cognitive impairment and LID with regard to dyskinesia control [69, 70]. No effect was found for riluzole on LID [71, 72]. In general, the high expectations that were raised with the potential therapeutic impact of antiglutamatergic drugs for PD have so far been disappointed.

#### 4.2.4. Drugs Acting on the Serotoninergic System

The serotoninergic system projects quite profusely to the striatum and also to other key basal ganglia nuclei (i.e., STN, GPe, GPi), exerting an inhibitory effect on dopamine striatal transmission. Durif et al. found a 47% improvement in LID severity induced by apomorphine in 7 patients with PD treated with fluoxetine [73], out of any reduction in antiparkinsonian benefits. Buspirone has a complex mechanism of action, which aside from its 5HT_1A_ properties includes partial dopamine agonism and mild opiate and noradrenergic antagonism [74]. Bonifati et al. in a double-blind, placebo-controlled, cross-over study, found that buspirone significantly lessened the severity of LID in 5 out of 7 patients [75]. Meco et al., in an open-label study including 20 parkinsonian patients, found that mirtazapine, an *α*_2_ antagonist, 5HT_1A_ agonist, and 5HT_2_ antagonist, may be effective in reducing LID [76].

#### 4.2.5. Drugs Acting on the Opioid System

The opioid striatal neurons may play a role in the induction of dyskinesias. In MPTP monkeys Samadi et al. investigated the effect of different doses of naloxone and naltrexone (opioid receptor antagonists) on the dyskinetic response to the D_1_ agonist SKF-82958, the D_2_ agonist quinpirole and levodopa [77]. They found that joint administration of naloxone or naltrexone together with dopaminergic agents led to a significant reduction in the severity of dyskinesias without reducing antiparkinsonian efficacy. Recently, the selective *μ* opioid antagonist ADL5510 provided almost complete alleviation of LID without compromising reversal of parkinsonian disability in the MPTP lesioned macaque model of PD [78]. In PD patients, Carroll et al. conducted a placebo-controlled, double-blind, cross-over trial to examine the potential effect of cannabis on LID in PD [79]. Seventeen patients completed the trial and cannabis was well tolerated with no pro- or anti-parkinsonian action, but there was no evidence of a treatment effect on LID. Thus, despite many experimental suggestions, there is no drug currently employed clinically to manipulate the opioid system for the treatment of LID. 

#### 4.2.6. Noradrenergic Drugs

The close relationship between the dopaminergic, adrenergic and noradrenergic systems has led to the assessment of a possible antidyskinetic effect of a few drugs acting on those systems. Carpentier et al. found a significant 40% improvement in dyskinesia scores in PD patients treated with a low dose of propranolol [80]. Other studies have shown how the *α*_2_ adrenoreceptor antagonist idazoxan can significantly reduce LID in monkey and rat models as well as in advanced PD patients [81, 82]. Rascol et al. reported improvement of LID without reappearance of parkinsonian symptoms in 18 patients treated with idazoxan [83]. Another *α*_2_ antagonist, fipamezole, reduced the severity of LID by 23% and 31% at 60 mg, and 90 mg respectively, without affecting antiparkinsonian response. Currently, further trials are being carried out [84].

#### 4.2.7. Adenosine A_2A_ Antagonists

Adenosine A_2A_ receptors are found in the striatum and thalamus and colocalize with dopamine D_2_ receptors. Adenosine A_2A_ antagonists regulate dopamine and glutamate release in the brain, and they may improve motor symptoms as novel compensatory mode for loss of dopamine signaling with associated NMDA antagonism [85]. The trials target symptoms associated with dopamine replacement and therapy of dyskinesia, such as istradefylline [86, 87]. However, recent trial outcomes showed that istradefylline did not improve motor behavior or “off” times in PD patients compared with earlier results [88–92]. Preladenant showed, in a phase II placebo-controlled dose-ranging trial of 253 PD patients receiving stable dopaminergic therapy, an increase in awake time spent in the on-state of 1.4 h/day compared to 0.2 h/day in the placebo group, without overall worsening of dyskinesias [93]. The long-term antidyskinetic effect of preladenant needs ascertainment.

#### 4.2.8. Other Drug Treatments

Levetiracetam, an antiepileptic drug, has been evaluated against LID with mixed results in several open-label studies [94–98]. The most promising data come from a study of 9 patients experiencing LID for at least 25% of waking hours [98]. After 60 days treatment with a mean of 625 mg of levetiracetam, patients experienced a 42% increase in the “on” time without LID or with nontroublesome dyskinesia in absence of significant change in the “off” time. Pardoprunox is a mixed dopamine agonist/antagonist D_2_ and D_3_, and a full agonist at 5HT_1a_ receptors. It also binds with lower affinity to D_4_, *α*_1_ adrenergic, and 5HT_7_ receptors [99, 100]. Due to its unique pharmacologic profile, pardoprunox might have a lower tendency than other dopaminergic therapies to cause dyskinesias or neuropsychiatric side effects [93, 99–101]. Safinamide is an antiparkinsonian agent that is also in advace state of development to reach clinical practice. It has a dual mechanism of action, as it is a MAO_B_ inhibitor and also reduces overactivity of glutamatergic signaling by inhibiting glutamate release [102, 103]. On this prospection, AFQ056 recently achieved a significant and relevant antidyskinetic clinical effect without reducing the antiparkinsonian benefits of dopaminergic therapy [104]. Recently, low-frequency transcranial magnetic stimulation has also been applied to the treatment of LID, showing transient experimental improvements in preliminary study [105].

#### 4.2.9. Practical Considerations

There appear to be many drugs that are capable of reducing LID severity. In occasional patients the therapeutic impact of any one of the treatments summarized above may be strikingly positive, but in the majority of patients it is limited to mild and short-lasting improvement. Nevertheless, these treatments are generally well tolerated and worth trying, when available, in patients in whom other therapeutic measurements cannot be afforded. In our experience, the degree of symptomatic control of LID mainly depends upon the complexity of dyskinesias and severity of “off” periods. This may be schematically summarized as follows: (1) in patients with mild but bothersome peak-dose dyskinesias, readjust the levodopa schedule, and consider adding a dopamine agonist. If this approach fails, any one of the drugs discussed above may be tried out; (2) for patients with intense peak-dose dyskinesias, consider switching treatment to provide continuous dopaminergic stimulation; (3) patients with severe peak-dose dyskinesias and diphasic dyskinesias probably require surgical treatment (Table 1).

### 4.3. Continuous Dopaminergic Stimulation

Since the introduction of the concept of continuous dopaminergic stimulation in the 1980s [3, 106–108], it has been realized that constant delivery of dopaminergic drugs is associated with a reduction in LID severity. Over the past decade, further evidence has accumulated to support the notion that continuous stimulation of dopamine receptors may even reverse some of the changes induced by chronic pulsatile levodopa administration. The antidyskinetic response to this approach is not immediate and it may take several weeks of continuous infusion before becoming apparent. The initial pivotal study using continuous delivery was published by Mouradian et al., who used levodopa intravenously for 7–12 days to a small group (*n* = 12) of patients with advanced PD [109]. They found a progressive attenuation of LID and improvement of the “on–off” fluctuations. Levodopa is too acid to be delivered intravenously or subcutaneously in practice, a problem by and large resolved with the development of duodenal levodopa infusion. This has been used with clear benefit to improve motor complications and quality of life despite the obvious practical limitations [110–113]. Very recently, the first double-blind, placebo controlled study assessing the effect of duodenal levodopa carried out in North America has been disclosed. However, the technique is complex, expensive, and potential long-term adverse effects are under debate, such as axonal polyneuropathy and vitamin B complex deficiency [114, 115]. The infusion of the duodenal levodopa gel, which also contains the dopa-decarboxylase inhibitor carbidopa, is currently available only in certain countries.

Further alternative strategies of oral intake were also tested, such as controlled release levodopa/carbidopa formulations, but they did not delay the onset of motor complications [116]. The STRIDE-PD study, initiating levodopa with entacapone, failed to reduce the frequency or delay the onset of LID [117]; an inadequate dosing schedule perturbing the putative continuous stimulation expected to be achieved with this treatment and a bias in the treatment group toward more severe disease have been suggested as potential confounders [118]. IPX066 might be soon available and it may be used to attain and maintain therapeutic levodopa plasma concentrations with a potential antidyskinetic efficacy [119].

In line with continuous delivery procedures, dopamine agonists that operate via the subcutaneous route, such as lisuride and apomorphine, are associated with a reduction in LID. The majority of trials used infusions during the daytime but stopped at night to reduce the risk of severe psychiatric complications. Stocchi et al. compared the long-term incidence of dyskinesias in patients treated with subcutaneous infusion of lisuride (plus supplementary oral levodopa as needed) versus patients treated with standard levodopa orally, and showed that patients receiving lisuride infusions experienced a reduction in the incidence of dyskinesia and motor fluctuations, compared with patients receiving standard therapies [120]. The benefit lasted over the 4 years of follow-up and this study also endorsed earlier results indicating that continuous lisuride infusion can be fairly well tolerated and beneficial for patients' motor complications, provided they have not previously developed severe psychiatric complications [121, 122]. 

Similarly, Manson et al. reviewed their experience in 64 patients treated with subcutaneous apomorphine infusions [123]. Forty-five patients were successfully converted to monotherapy and discontinued all other dopaminergic drugs during the daytime infusion. LID were reduced by 64% in the monotherapy group compared to 30% in those on polytherapy. Another retrospective evaluation over a 5-year period of 82 patients receiving apomorphine obtained a similar outcome [124], with average follow-up of *≈*20 months, 5 mg/h dose, and 14 hours/day duration. Patients improved in severity of dyskinesia by 31% as assessed by the UPDRS dyskinesia evaluation, injection-site adverse events being the main reason for discontinuation of treatment. These results confirmed that monotherapy with infusions of apomorphine may reset peak-dose dyskinesia threshold in patients treated with levodopa, while further reducing off-period disability. Katzenschlager et al. prospectively assessed the antidyskinetic effect of continuous subcutaneous apomorphine using subjective and objective measures and response to a levodopa challenge [125]. By the sixth month the mean levodopa dose had been reduced by 55% and the daily “off” time in patients' diaries was reduced by 38%. Levodopa challenge showed a reduction of 40–44% in the dyskinesia scores and patients' self-assessment scores reflected these significant changes positively. Overall, these results reinforce the concept that replacement of oral short-acting antiparkinsonian drugs with medication capable of providing more continuous dopamine receptor stimulation may at least partially avoid or reverse the sensitization process believed to mediate the development of LID. In theory, therefore, therapy with infusions capable of providing continuous dopaminergic stimulation might be the pharmacological treatment of choice for advanced PD patients. Nevertheless, the degree of control of LID achieved with infusions is not complete in many patients. Pharmacological tolerance appears in a large proportion after some time on treatment. It occurs more readily the more severe the underlying disease is, leading to “off” episodes or exacerbation of diphasic dyskinesias. The latter may cause a very troublesome dyskinetic status [122]. At this point, surgical treatment may still be the only and best therapeutic option for a proportion of patients with severe LID.

## 5. Surgical Treatments

The three main surgical targets for PD are the thalamus, GPi, and STN. In this section we review the antidyskinetic effect of stereotactic surgery directed towards these 3 different targets using either ablative surgery or DBS.

### 5.1. The Thalamus and LID

#### 5.1.1. Vim-Thalamotomy

During the 1960s the ventral lateral nucleus (VL) of the thalamus was determined as the best target to remove tremor in PD. This target was later defined from physiology as the ventralis intermedius (Vim) and it became established as the target of choice for tremor of any origin [126–128]. Despite the thousands of thalamotomies performed over the years, no formal and prospective evaluation of the response of thalamic surgery against LID has been reported in the literature. Some reports described how the development of LID was prevented in patients with a previous thalamotomy [129–132], others described how the lesion improved tremor and also LID [133–135], or the concept that LID improvement should be correlated with daily levodopa reduction after surgery following tremor suppression [136]. However, there are also reports where no benefit was obtained with Vim-thalamotomy in patients with LID [137].

The best study, which is somewhat an exception to the above, is the observational paper published by Narabayashi et al. [138]. These authors report an interesting and detailed study of the effect of thalamotomy against LID, dividing the patients according to the thalamic target selected for surgery. The patients subjected to lesions in the ventralis oralis anterior nucleus (Voa) or posterior (Vop) prior to the introduction of levodopa did not develop LID, but patients subjected to Vim-thalamotomy for tremor did develop dyskinesias when levodopa was introduced as treatment [138]. The conclusion reached by Narabayashi et al. was that the GPi-Voa/Vop pathway mediated LID and lesions restricted to the Vim to treat tremor were not effective against LID. Interestingly, similar results were reported by Page et al. in parkinsonian monkeys with LID induced by dopamine agonists. Thalamotomy performed in the pallidal territory removed LID, but lesions in the nigral or cerebellar terminal territory of the thalamus had no antidyskinetic effect [139].

#### 5.1.2. Vim-DBS

The introduction of high frequency stimulation coupled with stereotactic surgery supposed a marked advance for patients with movement disorders. Vim-DBS was initially performed as an additional contralateral treatment to patients who had had a previous thalamotomy [140]. In a group of parkinsonian patients, Benabid et al. described significant tremor improvement after Vim-DBS, which was accompanied by inconsistent responses or no alleviation of LID [141]. Similar results were obtained in other studies [142–144]. In contrast, successful alleviation or suppression of LID was described in association with a different positioning of the electrode which supposedly impinges upon the Vim and the Centromedian-parafascicular nucleus (CM-Pf) [145–147]. However, a more recent study in MPTP treated monkeys revealed that lesion of the CM-Pf had no effect against parkinsonian features or LID [148]. In conclusion, the available data indicate that Vim lies outside the pathways underlying LID and, accordingly, Vim's surgery conveys no effect against LID.

### 5.2. Surgery of the GPi and LID

#### 5.2.1. Pallidotomy

Posteroventral pallidotomy was reintroduced as a treatment for PD, applying Leksell's concepts, by Laitinen et al. in 1992 [149]. The clinical response to pallidal lesion included a significant benefit of the cardinal features on the contralateral side and, unexpectedly according to the basal ganglia model, a large impact against LID. Thus, pallidotomy has been shown to portray a very significant and long lasting effect against peak dose dyskinesia, diphasic dyskinesia, and also “off” period dystonia on the side contralateral to the lesion. This antidyskinetic effect is enduring and long-lasting, for at least 10 years [150, 151], with a benefit that occurred without a significant reduction in daily levodopa dose.

#### 5.2.2. GPi-DBS and LID

In the first multicentre DBS Cooperative Multicentre Study after GPi-DBS, patients showed a 76% reduction in LID severity (*P* < 0.0001) with no change in levodopa doses at 1 and 4 years follow up [152, 153]. Longer follow-up (5-6 years) continued to show that GPi-DBS maintained a significant improvement of LID with a significant increase in “on” time without LID [154]. Levodopa was not significantly reduced compared with baseline [155].

### 5.3. STN Surgery and LID

#### 5.3.1. Subthalamotomy

The STN plays a capital role in the pathophysiology of parkinsonian and dyskinetic states. This anatomical target is typically considered a prodyskinetic structure and classically avoided in patients with severe LID. Subthalamotomy is performed on occasional patients, more frequently in countries where DBS is not affordable, with fairly good general results [156].

Assessing the evolution of LID after subthalamotomy is limited by the relatively reduced number of patients reported, and by the variables in controlling some important factors, such as levodopa dose pre- and postsurgery, surgical procedure, lesion placement and volume. A recent analysis described how in a group of 68 patients “peak dose dyskinesias” increased on the side contralateral to the lesion during the first postoperative year but decreased after two to three years, showing no significant change versus baseline at the last assessment. In the ipsilateral side to the lesion, LID increased significantly with the progressive increment of levodopa suggesting that the operated side has had an antidyskinetic effect. Diphasic dyskinesias and “off” period dystonia also improved significantly (*P* < 0.01) contralateral to the lesion at 12th and 24th months after surgery [156].

#### 5.3.2. STN-DBS

Bilateral STN-DBS is currently the surgical procedure most often selected for PD patients given the large impact against “off” medication severity and the associated reduction in the daily levodopa dose [153, 157–162]. STN-DBS has generally been associated with significant reduction in LID, closely correlated with levodopa dosage reduction. Subthalamic stimulation appears to improve the whole spectrum of LID, such as peak dose dyskinesia (30%), biphasic dyskinesia (50%), and “off” dystonia (90%) with a 47% reduction in levodopa dosage as reported by Krack et al. [157]. DBS-STN also increases “on” time without LID and reduces “off” time periods [161–163]. After 5-6 years of follow-up, LID scores were significantly improved by 83.3% in total, with 75% reduction in dyskinesia duration and 100% drop of disability compared with baseline [153, 155, 161]. Levodopa reduction was also significantly reduced in the long term compared with baseline preoperative data (30%) [155]. In a survey of 38 studies involving 737 patients treated in 34 neurosurgical centers, STN-DBS improved LID assessed by UPDRS-IV scores 94% at 12 months in the on-stimulation/“on” medication state in comparison with “on” preoperative medication scores [163].

How STN-DBS may improve LID is not well understood [164]. For most authors, LID improvement by STN-DBS may be directly correlated with levodopa reduction [165–169]. However, it is difficult to interpret these studies, because there are very few patients who maintained similar levodopa equivalent doses after surgery. Thus, fluctuations and LID disappeared in patients with levodopa withdrawal postimplantation as Vingenhoerst et al. described, whereas they persisted in those patients on medication 2 years after surgery [167]. Similarly, another group reported that 1 year after implantation, patients receiving levodopa displayed a 47% LID reduction, whereas the reduction was 90% of LID in patients who did not receive levodopa (*P* < 0.003) [168]. On the other hand, the antidyskinetic response after STN-DBS could be related with the effect of continuous high frequency stimulation, providing antidyskinetic efficacy on its own [170–172]. This may be supported by some instances where improvement of LID occurred despite maintaining the same daily dose of levodopa [170]. Thus, STN surgery could induce a stable and continuous functional state with reduced fluctuations in basal ganglia network, somehow mimicking the effect of continous dopaminergic stimulation.

Finally, it has also been suggested that the antidyskinetic effect of STN-DBS (as well as subthalamotomy) may be due to an effect on the dorsal border of the nucleus, reaching the lenticularis fasciculus and zona incerta. In this context some studies have suggested that the real subthalamic target may be the region above the dorsal border of the nucleus [173–175].

In conclusion, STN-DBS probably interferes with abnormal discharge pattern in basal ganglia output nuclei associated with the parkinsonian condition, improving PD, and permitting a reduction of chronic levodopa therapy. The latter is likely responsible for the anti-LID effect. On the other hand, it is also possible that high frequency stimulation of the STN could modify the patterns of neuronal firing and the rhythms associated with LID having “per se” an anti-dyskinetic effect [176].

## 6. Conclusions

Most PD patients develop motor fluctuations and LID during chronic evolution and on levodopa treatment. Motor complications are directly related with disease progression and the effects of chronic levodopa therapy. Once established, LID remains unabated throughout evolution. Pharmacological management is not simple but in recent years, the proportion of patients suffering severe LID has declined considerably, mainly in relation with the use smaller dose of levodopa. Surgical treatment has a potent anti-dyskinetic effect whose value has to be judged for every particular patient against the risk. LID is no longer the major cause of disability in PD patients nor a problem lacking several treatment options.

## Figures and Tables

**Table 1:** 

Practical suggestions for pharmacological management of LID
(1) The optimal therapeutic approach for LID is to try avoiding their development
(2) Start PD treatment with an agonist if possible, particularly in young onset patients
(3) Save levodopa as long as you can hold the patient's requirements for daily life activities
(4) Adjust the drug schedule: reduce total daily doses and/or shorten the intake intervals
(5) Add amantadine 200–400 mg/day
(6) Low doses of quetiapine or clozapine may be helpful
(7) Propose continuous drug delivery devices: duodenal levodopa/carbidopa gel or subcutaneous apomorphine
(8) For refractory cases, when indication is set by an expert and the risks are assumable by the patient, surgery is the treatment of choice

## References

[1] Barbeau A (1975). Long term assessment of levodopa therapy in Parkinson’s disease. *Canadian Medical Association Journal*.

[2] Marsden CD, Parkes JD (1977). Success and problems of long term levodopa therapy in Parkinson’s disease. *The Lancet*.

[3] Obeso JA, Grandas F, Vaamonde J (1989). Motor complications associated with chronic levodopa therapy in Parkinson’s disease. *Neurology*.

[4] Luquin MR, Scipioni O, Vaamonde J, Gershanik O, Obeso JA (1992). Levodopa-induced dyskinesias in Parkinson’s disease: clinical and pharmacological classification. *Movement Disorders*.

[5] Rascol O, Brooks DJ, Korczyn AD, De Deyn PP, Clarke CE, Lang AE (2000). A five-year study of the incidence of dyskinesia in patients with early Parkinson’s disease who were treated with ropinirole or levodopa. *The New England Journal of Medicine*.

[6] Obeso JA, Rodriguez-Oroz MC, Rodriguez M, DeLong MR, Olanow CW (2000). Pathophysiology of levodopa-induced dyskinesias in Parkinson’s disease: problems with the current model. *Annals of Neurology*.

[7] Růžička E, Zárubová K, Nutt JG, Bloem BR (2011). ‘Silly Walks’ in Parkinson’s disease: unusual presentation of dopaminergic-induced dyskinesias. *Movement Disorders*.

[8] Freed CR, Greene PE, Breeze RE (2001). Transplantation of embryonic dopamine neurons for severe Parkinson's disease. *The New England Journal of Medicine*.

[9] Limousin P, Krack P, Pollak P (1998). Electrical stimulatiion of the subthalamic nucleus in advanced Parekinson's disease. *The New England Journal of Medicine*.

[10] Olanow CW, Kordower JH, Lang AE, Obeso JA (2009). Dopaminergic transplantation for Parkinson’s disease: current status and future prospects. *Annals of Neurology*.

[11] Olanow CW, Gracies JM, Goetz CG (2009). Clinical pattern and risk factors for dyskinesias following fetal nigral transplantation in parkinson’s disease: a double blind video-based analysis. *Movement Disorders*.

[12] Olanow CW, Schapira AHV, Rascol O (2000). Continuous dopamine-receptor stimulation in early Parkinson’s disease. *Trends in Neurosciences*.

[13] Cenci MA, Lee CS, Björklund A (1998). L-DOPA-induced dyskinesia in the rat is associated with striatal overexpression of prodynorphin- and glutamic acid decarboxylase mRNA. *European Journal of Neuroscience*.

[14] Calabresi P, Filippo MD, Ghiglieri V, Tambasco N, Picconi B (2010). Levodopa-induced dyskinesias in patients with Parkinson’s disease: filling the bench-to-bedside gap. *The Lancet Neurology*.

[15] Filion M, Tremblay L, Bedard PJ (1991). Effects of dopamine agonists on the spontaneous activity of globus pallidus neurons in monkeys with MPTP-induced parkinsonism. *Brain Research*.

[16] Papa SM, Desimone R, Fiorani M, Oldfield EH (1999). Internal globus pallidus discharge is nearly suppressed during levodopa-induced dyskinesias. *Annals of Neurology*.

[17] Levy R, Dostrovsky JO, Lang AE, Sime E, Hutchison WD, Lozano AM (2001). Effects of apomorphine on subthalamic nucleus and globus pallidus internus neurons in patients with Parkinson’s disease. *Journal of Neurophysiology*.

[18] Lozano AM, Lang AE, Levy R, Hutchison W, Dostrovsky J (2000). Neuronal recordings in Parkinson’s disease patients with dyskinesias induced by apomorphine. *Annals of Neurology*.

[19] Merello M, Balej J, Delfino M, Cammarota A, Betti O, Leiguarda R (1999). Apomorphine induces changes in GPi spontaneous outflow in patients with Parkinson's disease. *Movement Disorders*.

[20] Mitchell IJ, Boyce S, Sambrook MA, Crossman AR (1992). A 2-deoxyglucose study of the effects of dopamine agonists on the Parkinsonian primate brain. Implications for the neural mechanisms that mediate dopamine agonist-induced dyskinesia. *Brain*.

[21] Albin RL (1995). The pathophysiology of chorea/ballism and Parkinsonism. *Parkinsonism and Related Disorders*.

[22] Albin RL, Young AB, Penney JB (1989). The functional anatomy of basal ganglia disorders. *Trends in Neurosciences*.

[23] Crossman AR (1987). Primate models of dyskinesia: the experimental approach to the study of basal ganglia-related involuntary movement disorders. *Neuroscience*.

[24] DeLong MR (1990). Primate models of movement disorders of basal ganglia origin. *Trends in Neurosciences*.

[25] Crossman AR, Mitchell IJ, Sambrook MA, Jackson A (1988). Chorea and myoclonus in the monkey induced by gamma-aminobutyric acid antagonism in the lentiform complex. The site of drug action and a hypothesis for the neural mechanisms of chorea. *Brain*.

[26] Hamada I, DeLong MR (1992). Excitotoxic acid lesions of the primate subthalamic nucleus result in transient dyskinesias of the contralateral limbs. *Journal of Neurophysiology*.

[27] Mitchell IJ, Jackson A, Sambrook MA, Crossman AR (1989). The role of the subthalamic nucleus in experimental chorea. Evidence from 2-deoxyglucose metabolic mapping and horseradish peroxidase tracing studies. *Brain*.

[28] Berthet A, Bezard E, Porras G (2012). L-DOPA impairs proteasome activity in parkinsonism through D_1_ dopamine receptor. *Journal of Neuroscience*.

[29] Iravani MM, McCreary AC, Jenner P (2012). Striatal plasticity in Parkinson's disease and L-DOPA induced dyskinesia. *Parkinsonism and Related Disorders*.

[30] Aubert I, Guigoni C, Håkansson K (2005). Increased D_1_ dopamine receptor signaling in levodopa-induced dyskinesia. *Annals of Neurology*.

[31] Bordet R, Ridray S, Schwartz JC, Sokoloff P (2000). Involvement of the direct striatonigral pathway in levodopa-induced sensitization in 6-hydroxydopamine-lesioned rats. *European Journal of Neuroscience*.

[32] Guigoni C, Dovero S, Aubert I (2005). Levodopa-induced dyskinesia in MPTP-treated macaques is not dependent on the extent and pattern of nigrostrial lesioning. *European Journal of Neuroscience*.

[33] Picconi B, Centonze D, Håkansson K (2003). Loss of bidirectional striatal synaptic plasticity in L-DOPA-induced dyskinesia. *Nature Neuroscience*.

[34] Berthet A, Porras G, Doudnikoff E (2009). Pharmacological analysis demonstrates dramatic alteration of D_1_ dopamine receptor neuronal distribution in the rat analog of L-DOPA-induced dyskinesia. *Journal of Neuroscience*.

[35] Bézard E, Ferry S, Mach U (2003). Attenuation of levodopa-induced dyskinesia by normalizing dopamine D_3_ receptor function. *Nature Medicine*.

[36] Fiorentini C, Busi C, Gorruso E, Gotti C, Spano P, Missale C (2008). Reciprocal regulation of dopamine D_1_ and D_3_ receptor function and trafficking by heterodimerization. *Molecular Pharmacology*.

[37] Smith Y, Raju D, Nanda B, Pare JF, Galvan A, Wichmann T (2009). The thalamostriatal systems: anatomical and functional organization in normal and parkinsonian states. *Brain Research Bulletin*.

[38] Villalba RM, Smith Y (2011). Neuroglial plasticity at striatal glutamatergic synapses in Parkinson's disease. *Frontiers in Systems Neuroscience*.

[39] Gardoni F, Picconi B, Ghiglieri V (2006). A critical interaction between NR2B and MAGUK in L-DOPA induced dyskinesia. *Journal of Neuroscience*.

[40] Bateup HS, Santini E, Shen W (2010). Distinct subclasses of medium spiny neurons differentially regulate striatal motor behaviors. *Proceedings of the National Academy of Sciences of the United States of America*.

[41] Ghiglieri V, Picconi B, Calabresi P (2010). Direct and indirect pathways in levodopa-induced dyskinesia: a more complex matter than a network imbalance. *Movement Disorders*.

[42] Belujon P, Lodge DJ, Grace AA (2010). Aberrant striatal plasticity is specifically associated with dyskinesia following levodopa treatment. *Movement Disorders*.

[43] Aron AR, Obeso J (2012). Is executive control used to compensate for involuntary movements in levodopa-induced dyskinesia?. *Movement Disorders*.

[44] Shoulson I (1998). DATATOP: a decade of neuroprotective inquiry. *Annals of Neurology*.

[45] Rascol O, Brooks DJ, Korczyn AD (2006). Development of dyskinesias in a 5-year trial and ropinirole and L-dopa. *Movement Disorders*.

[46] Hauser RA, Rascol O, Korczyn AD (2007). Ten-year follow-up of Parkinson’s disease patients randomized to initial therapy with ropinirole or levodopa. *Movement Disorders*.

[47] Watts RL, Lyons KE, Pahwa R (2010). Onset of dyskinesia with adjunct ropinirole prolonged-release or additional levodopa in early Parkinson’s disease. *Movement Disorders*.

[48] Maratos EC, Jackson MJ, Pearce RKB, Jenner P (2001). Antiparkinsonian activity and dyskinesia risk of ropinirole and L-DOPA combination therapy in drug naive MPTP-lesioned common marmosets (*Callithrix jacchus*). *Movement Disorders*.

[49] Holloway R (2000). A randomized controlled trial comparing pramipexole with levodopa in early Parkinson’s disease: design and methods of the CALM-PD study. *Clinical Neuropharmacology*.

[50] Holloway R, Marek K, Biglan K (2009). Long-term effect of initiating pramipexole vs levodopa in early Parkinson disease. *Archives of Neurology*.

[51] Bracco F, Battaglia A, Chouza C (2004). The long-acting dopamine receptor agonist cabergoline in early Parkinson’s disease: final results of a 5-year, double-blind, levodopa-controlled study. *CNS Drugs*.

[52] Inzelberg R, Schechtman E, Nisipeanu P (2003). Cabergoline, pramipexole and ropinirole used as monotherapy in early Parkinson’s disease: an evidence-based comparison. *Drugs and Aging*.

[53] Pinero A, Marcos-Alberca P, Fortes J (2005). Cabergoline-related severe restrictive mitral regurgitation. *The New England Journal of Medicine*.

[54] Voon V, Fernagut PO, Wickens J (2009). Chronic dopaminergic stimulation in Parkinson’s disease: from dyskinesias to impulse control disorders. *The Lancet Neurology*.

[55] Bélanger N, Grégoire L, Tahar AH, Bédard PJ (2003). Chronic treatment with small does of cabergoline prevents dopa-induced dyskinesias in Parkinsonian monkeys. *Movement Disorders*.

[56] Pirtosek Z, Merello M, Carlsson A, Stern G (1993). Preclamol and parkinsonian fluctuations. *Clinical Neuropharmacology*.

[57] Lieberman JA (2004). Dopamine partial agonists: a new class of antipsychotic. *CNS Drugs*.

[58] Meco G, Stirpe P, Edito F (2009). Aripiprazole in l-dopa-induced dyskinesias: a one-year open-label pilot study. *Journal of Neural Transmission*.

[59] Oh JD, Bibbiani F, Chase TN (2002). Quetiapine attenuates levodopa-induced motor complications in rodent and primate parkinsonian models. *Experimental Neurology*.

[60] Katzenschlager R, Manson AJ, Evans A, Watt H, Lees AJ (2004). Low dose quetiapine for drug induced dyskinesias in Parkinson’s disease: a double blind cross over study. *Journal of Neurology, Neurosurgery and Psychiatry*.

[61] Durif F, Debilly B, Galitzky M (2004). Clozapine improves dyskinesias in Parkinson disease A double-blind, placebo-controlled study. *Neurology*.

[62] Metman LV, Del Dotto P, Blanchet PJ, Van Den Munckhof P, Chase TN (1998). Blockade of glutamatergic transmission as treatment for dyskinesias and motor fluctuations in Parkinson’s disease. *Amino Acids*.

[63] Del Dotto P, Pavese N, Gambaccini G (2001). Intravenous amantadine improves levadopa-induced dyskinesias: an acute double-blind placebo-controlled study. *Movement Disorders*.

[64] Luginger E, Wenning GK, Bösch S, Poewe W (2000). Beneficial effects of amantadine on L-dopa-induced dyskinesias in Parkinson’s disease. *Movement Disorders*.

[65] Snow BJ, Macdonald L, Mcauley D, Wallis W (2000). The effect of amantadine on levodopa-induced dyskinesias in Parkinson’s disease: a double-blind, placebo-controlled study. *Clinical Neuropharmacology*.

[66] Thomas A, Iacono D, Luciano AL, Armellino K, Di Iorio A, Onofrj M (2004). Duration of amantadine benefit on dyskinesia of severe Parkinson’s disease. *Journal of Neurology, Neurosurgery and Psychiatry*.

[67] Merello M, Perez-Lloret S, Antico J, Obeso JA (2006). Dyskinesias induced by subthalamotomy in Parkinson’s disease are unresponsive to amantadine. *Journal of Neurology, Neurosurgery and Psychiatry*.

[68] Merello M, Nouzeilles MI, Cammarota A, Leiguarda R (1999). Effect of memantine (NMDA antagonist) on Parkinson’s disease: a double-blind crossover randomized study. *Clinical Neuropharmacology*.

[69] Lökk J (2004). Memantine can relieve certain symptoms in Parkinson’s disease. *Lakartidningen*.

[70] Varanese S, Howard J, Di Rocco A (2010). NMDA antagonist memantine improves levodopa-induced dyskinesias and “on-off” phenomena in Parkinson’s disease. *Movement Disorders*.

[71] Braz CA, Borges V, Ferraz HB (2004). Effect of riluzole on dyskinesia and duration of the on state in Parkinson disease patients: a double-blind, placebo-controlled pilot study. *Clinical Neuropharmacology*.

[72] Bara-Jimenez W, Dimitrova TD, Sherzai A, Aksu M, Chase TN (2006). Glutamate release inhibition ineffective in levodopa-induced motor complications. *Movement Disorders*.

[73] Durif F, Vidailhet M, Bonnet AM, Blin J, Agid Y (1995). Levodopa-induced dyskinesias are improved by fluoxetine. *Neurology*.

[74] Kleedorfer B, Lees AJ, Stern GM (1991). Buspirone in the treatment of levodopa induced dyskinesias. *Journal of Neurology Neurosurgery and Psychiatry*.

[75] Bonifati V, Fabrizio E, Cipriani R, Vanacore N, Meco G (1994). Buspirone in levodopa-induced dyskinesias. *Clinical Neuropharmacology*.

[76] Meco G, Fabrizio E, Di Rezze S, Alessandri A, Pratesi L (2003). Mirtazapine in L-dopa-induced dyskinesias. *Clinical Neuropharmacology*.

[77] Samadi P, Grégoire L, Bédard PJ (2004). The opioid agonist morphine decreases the dyskinetic response to dopaminergic agents in parkinsonian monkeys. *Neurobiology of Disease*.

[78] Koprich JB, Fox SH, Johnston TH (2011). The selective mu-opioid receptor antagonist adl5510 reduces levodopa-induced dyskinesia without affecting antiparkinsonian action in mptp-lesioned macaque model of Parkinson’s disease. *Movement Disorders*.

[79] Carroll CB, Bain PO, Teare L (2004). Cannabis for dyskinesia in Parkinson disease: a randomized double-blind crossover study. *Neurology*.

[80] Carpentier AF, Bonnet AM, Vidailhet M, Agid Y (1996). Improvement of levodopa-induced dyskinesia by propranolol in Parkinson’s disease. *Neurology*.

[81] Buck K, Voehringer P, Ferger B (2010). The *α*2 adrenoceptor antagonist idazoxan alleviates l-DOPA-induced dyskinesia by reduction of striatal dopamine levels: an in vivo microdialysis study in 6-hydroxydopamine-lesioned rats. *Journal of Neurochemistry*.

[82] Grondin R, Tahar AH, Doan VD, Ladure P, Bédard PJ (2000). Noradrenoceptor antagonism with idazoxan improves L-dopa-induced dyskinesias in MPTP monkeys. *Naunyn-Schmiedeberg’s Archives of Pharmacology*.

[83] Rascol O, Arnulf I, Peyro-Saint Paul H (2001). Idazoxan, an alpha-2 antagonist, and L-DOPA-induced dyskinesias in patients with Parkinson’s disease. *Movement Disorders*.

[84] Gottwald MD, Aminoff MJ (2011). Therapies for dopaminergic-induced dyskinesias in Parkinson disease. *Annals of Neurology*.

[85] Müller T (2010). New small molecules for the treatment of Parkinson’s disease. *Expert Opinion on Investigational Drugs*.

[86] Brooks DJ, Papapetropoulos S, Vandenhende F (2010). An open-label, positron emission tomography study to assess adenosine A2A brain receptor occupancy of vipadenant (BIIB014) at steady-state levels in healthy male volunteers. *Clinical Neuropharmacology*.

[87] Jankovic J (2008). Are adenosine antagonists, such as istradefylline, caffeine, and chocolate, useful in the treatment of Parkinson’s disease?. *Annals of Neurology*.

[88] Pourcher E, Fernandez HH, Stacy M, Mori A, Ballerini R, Chaikin P (2012). Istradefylline for Parkinson's disease patients experiencing motor fluctuations: results of the KW-6002-US-018 study. *Parkinsonism and Related Disorders*.

[89] Fernandez HH, Greeley DR, Zweig RM, Wojcieszek J, Mori A, Sussman NM (2010). Istradefylline as monotherapy for Parkinson disease: results of the 6002-US-051 trial. *Parkinsonism and Related Disorders*.

[90] Hauser RA, Shulman LM, Trugman JM (2008). Study of istradefylline in patients with Parkinson’s disease on levodopa with motor fluctuations. *Movement Disorders*.

[91] Stacy M, Silver D, Mendis T (2008). A 12-week, placebo-controlled study (6002-US-006) of istradefylline in Parkinson disease. *Neurology*.

[92] LeWitt PA, Guttman M, Tetrud JW (2008). Adenosine A2A receptor antagonist istradefylline (KW-6002) reduces off time in Parkinson’s disease: a double-blind, randomized, multicenter clinical trial (6002-US-005). *Annals of Neurology*.

[93] Hauser RA, Cantillon M, Pourcher E (2011). Preladenant in patients with Parkinson’s disease and motor fluctuations: a phase 2, double-blind, randomised trial. *The Lancet Neurology*.

[94] Contin M, Martinelli P, Albani F (2007). Kinetic-dynamic monitoring of levetiracetam effects in patients with Parkinson disease and levodopa-induced dyskinesias. *Clinical Neuropharmacology*.

[95] Lyons KE, Pahwa R (2006). Efficacy and tolerability of levetiracetam in Parkinson disease patients with levodopa-induced dyskinesia. *Clinical Neuropharmacology*.

[96] Wolz M, Löhle M, Strecker K (2010). Levetiracetam for levodopa-induced dyskinesia in Parkinson’s disease: a randomized, double-blind, placebo-controlled trial. *Journal of Neural Transmission*.

[97] Wong KK, Alty JE, Goy AG, Raghav S, Reutens DC, Kempster PA (2011). A randomized, double-blind, placebo-controlled trial of levetiracetam for dyskinesia in Parkinson’s disease. *Movement Disorders*.

[98] Zesiewicz TA, Sullivan KL, Maldonado JL, Tatum WO, Hauser RA (2005). Open-label pilot study of levetiracetam (Keppra) for the treatment of levodopa-induced dyskinesias in Parkinson’s disease. *Movement Disorders*.

[99] Bronzova J, Sampaio C, Hauser RA (2010). Double-blind study of pardoprunox, a new partial dopamine agonist, in early Parkinson’s disease. *Movement Disorders*.

[100] Hauser RA, Bronzova J, Sampaio C (2009). Safety and tolerability of pardoprunox, a new partial dopamine agonist, in a randomized, controlled study of patients with advanced Parkinson’s disease for the pardoprunox study group. *European Neurology*.

[101] Rascol O, Bronzova J, Hauser RA (2012). Pardoprunox as adjunct therapy to levodopa in patients with Parkinson's disease experiencing motor fluctuations: results of a double-blind, randomized, placebo-controlled, trial. *Parkinsonism and Related Disorders*.

[102] Stocchi F, Arnold G, Onofrj M (2004). Improvement of motor function in early Parkinson disease by safinamide. *Neurology*.

[103] Grosset D, Grosset A (2010). The movement disorder society—14th international congress of Parkinson’s disease and movement disorders. *IDrugs*.

[104] Berg D, Godau J, Trenkwalder C (2011). AFQ056 treatment of levodopa-induced dyskinesias: results of 2 randomized controlled trials. *Movement Disorders*.

[105] Fregni F, Simon DK, Wu A, Pascual-Leone A (2005). Non-invasive brain stimulation for Parkinson’s disease: a systematic review and meta-analysis of the literature. *Journal of Neurology, Neurosurgery and Psychiatry*.

[106] Chase TN, Baronti F, Fabbrini G, Heuser IJ, Juncos JL, Mouradian MM (1989). Rationale for continuous dopaminomimetic therapy of Parkinson’s disease. *Neurology*.

[107] Horowski R, Marsden CD, Obeso JA (1988). Continuous dopaminergic stimulation: state of the art and outlook. *Journal of Neural Transmission, Supplement*.

[108] Obeso JA, Grandas F, Herrero MT, Horowski R (1994). The role of pulsatile versus continuous dopamine receptor stimulation for functional recovery in Parkinson’s disease. *European Journal of Neuroscience*.

[109] Mouradian MM, Heuser IJE, Baronti F, Chase TN (1990). Modification of central dopaminergic mechanisms by continuous levodopa therapy for advanced Parkinson’s disease. *Annals of Neurology*.

[110] Antonini A, Isaias IU, Canesi M (2007). Duodenal levodopa infusion for advanced Parkinson’s disease: 12-month treatment outcome. *Movement Disorders*.

[111] Devos D (2009). Patient profile, indications, efficacy and safety of duodenal levodopa infusion in advanced Parkinson's disease. *Movement Disorders*.

[112] Nyholm D, Lewander T, Johansson A, LeWitt PA, Lundqvist C, Aquilonius SM (2008). Enteral levodopa/carbidopa infusion in advanced Parkinson disease: long-term exposure. *Clinical Neuropharmacology*.

[113] Nyholm D, Nilsson Remahl AIM, Dizdar N (2005). Duodenal levodopa infusion monotherapy vs oral polypharmacy in advanced Parkinson disease. *Neurology*.

[114] Müller T, Renger K, Kuhn W (2004). Levodopa-associated increase of homocysteine levels and sural axonal neurodegeneration. *Archives of Neurology*.

[115] Onofrj M, Bonanni L, Cossu G, Manca D, Stocchi F, Thomas A (2009). Emergencies in parkinsonism: akinetic crisis, life-threatening dyskinesias, and polyneuropathy during L-Dopa gel treatment. *Parkinsonism and Related Disorders*.

[116] Block G, Liss C, Reines S (1997). Comparison of immediate-release and controlled release carbidopa/levodopa in Parkinson's disease. A multicenter 5-year study. *European Neurology*.

[117] Stocchi F, Rascol O, Kieburtz K (2010). Initiating levodopa/carbidopa therapy with and without entacapone in early Parkinson disease: the STRIDE-PD study. *Annals of Neurology*.

[118] Stoessl AJ (2010). Continuous dopaminergic therapy in Parkinson disease: time to stride back?. *Annals of Neurology*.

[119] Hauser RA, Ellenbogen AL, Metman LV (2011). Crossover comparison of IPX066 and a standard levodopa formulation in advanced Parkinson’s disease. *Movement Disorders*.

[120] Stocchi F, Ruggieri S, Vacca L, Olanow CW (2002). Prospective randomized trial of lisuride infusion versus oral levodopa in patients with Parkinson’s disease. *Brain*.

[121] Obeso JA, Luquin MR, Martinez-Lage JM (1986). Lisuride infusion pump: a device for the treatment of motor fluctuations in Parkinson’s disease. *The Lancet*.

[122] Vaamonde J, Luquin MR, Obeso JA (1991). Subcutaneous lisuride infusion in Parkinson’s disease. Response to chronic administration in 34 patients. *Brain*.

[123] Manson AJ, Turner K, Lees AJ (2002). Apomorphine monotherapy in the treatment of refractory motor complications of Parkinson’s disease: long-term follow-up study of 64 patients. *Movement Disorders*.

[124] García Ruiz PJ, Ignacio ÁS, Pensado BA (2008). Efficacy of long-term continuous subcutaneous apomorphine infusion in advanced Parkinson's disease with motor fluctuations: a multicenter study. *Movement Disorders*.

[125] Katzenschlager R, Hughes A, Evans A (2005). Continuous subcutaneous apomorphine therapy improves dyskinesias in Parkinson’s disease: a prospective study using single-dose challenges. *Movement Disorders*.

[126] Ohye C, Maeda T, Narabayashi H (1976). Physiologically defined VIM nucleus. Its special reference to control of tremor. *Applied Neurophysiology*.

[127] Lenz FA, Kwan HC, Martin RL, Tasker RR, Dostrovsky JO, Lenz YE (1994). Single unit analysis of the human ventral thalamic nuclear group. Tremor-related activity in functionally identified cells. *Brain*.

[128] Tasker RR, Kiss ZHT (1995). The role of the thalamus in functional neurosurgery. *Neurosurgery Clinics of North America*.

[129] Van Buren JM, Li CL, Shapiro DY (1973). A qualitative and quantitative evaluation of parkinsonians three to six years following thalamotomy. *Confinia Neurologica*.

[130] Husby J, Korsgaard AG (1975). Proceedings: late results of thalamotomy in Parkinsonism with and without the influence of levodopa. *Acta Neurochirurgica*.

[131] Derome PJ, Jedynak CP, Visot A, Delalande O (1986). Treatment of abnormal movements by thalamic lesions. *Revista de Neurología*.

[132] Nagaseki Y, Shibazaki T, Hirai T (1986). Long-term follow-up results of selective VIM-thalamotomy. *Journal of Neurosurgery*.

[133] Fox MW, Ahlskog JE, Kelly PJ (1991). Stereotactic ventrolateralis thalamotomy for medically refractory tremor in post-levodopa era Parkinson’s disease patients. *Journal of Neurosurgery*.

[134] Jankovic J, Cardoso F, Grossman RG, Hamilton WJ, Tasker RR, Kelly PJ (1995). Outcome after stereotactic thalamotomy for parkinsonian, essential, and other types of tremor. *Neurosurgery*.

[135] Kelly PJ, Gillingham FJ (1980). The long-term results of stereotaxic surgery and L-dopa therapy in patients with Parkinson’s disease. A 10-year follow-up study. *Journal of Neurosurgery*.

[136] Miyamoto T, Bekku H, Moriyama E, Tsuchida S (1985). Present role of stereotactic thalamotomy for Parkinsonism. Retrospective analysis of operative results and thalamic lesions in computed tomograms. *Applied Neurophysiology*.

[137] Diederich N, Goetz CG, Stebbins GT (1992). Blinded evaluation confirms long-term asymmetric effect of unilateral thalamotomy or subthalamotomy on tremor in Parkinson’s disease. *Neurology*.

[138] Narabayashi H, Yokochi F, Nakajima Y (1984). Levodopa-induced dyskinesia and thalamotomy. *Journal of Neurology Neurosurgery and Psychiatry*.

[139] Page RD, Sambrook MA, Crossman AR (1993). Thalamotomy for the alleviation of levodopa-induced dyskinesia: experimental studies in the 1-methyl-4-phenyl-1,2,3,6-tetrahydropyridine-treated Parkinsonian monkey. *Neuroscience*.

[140] Benabid AL, Pollak P, Hommel M, Gaio JM, de Rougemont J, Perret J (1989). Treatment of Parkinson tremor by chronic stimulation of the ventral intermediate nucleus of the thalamus. *Revista de Neurología*.

[141] Benabid AL, Pollak P, Seigneuret E, Hoffmann D, Gay E, Perret J (1993). Chronic VIM thalamic stimulation in Parkinson’s disease, essential tremor and extra-pyramidal dyskinesias. *Acta Neurochirurgica, Supplement*.

[142] Limousin P, Speelman JD, Gielen F, Janssens M (1999). Multicentre European study of thalamic stimulation in parkinsonian and essential tremor. *Journal of Neurology Neurosurgery and Psychiatry*.

[143] Hariz MI, Krack P, Alesch F (2008). Multicentre European study of thalamic stimulation for parkinsonian tremor: a 6 year follow-up. *Journal of Neurology, Neurosurgery and Psychiatry*.

[144] Tasker RR, Munz M, Junn FS (1997). Deep brain stimulation and thalamotomy for tremor compared. *Acta Neurochirurgica. Supplement*.

[145] Blond S, Caparros-Lefebvre D, Parker F (1992). Control of tremor and involuntary movement disorders by chronic stereotactic stimulation of the ventral intermediate thalamic nucleus. *Journal of Neurosurgery*.

[146] Caparros-Lefebvre D, Blond S, Vermersch P, Pecheux N, Guieu JD, Petit H (1993). Chronic thalamic stimulation improves tremor and levodopa induced dyskinesias in Parkinson’s disease. *Journal of Neurology Neurosurgery and Psychiatry*.

[147] Caparros-Lefebvre D, Blond S, Feltin MP, Pollak P, Benabid AL (1999). Improvement of levodopa induced dyskinesias by thalamic deep brain stimulation is related to slight variation in electrode placement: possible involvement of the centre median and parafascicularis complex. *Journal of Neurology Neurosurgery and Psychiatry*.

[148] Lanciego JL, Rodríguez-Oroz MC, Blesa FJ (2008). Lesion of the centromedian thalamic nucleus in MPTP-treated monkeys. *Movement Disorders*.

[149] Laitinen LV, Bergenheim AT, Hariz MI (1992). Leksell’s posteroventral pallidotomy in the treatment of Parkinson’s disease. *Journal of Neurosurgery*.

[150] Hariz MI, Bergenheim AT (2001). A 10-year follow-up review of patients who underwent Leksell’s posteroventral pallidotomy for Parkinson disease. *Journal of Neurosurgery*.

[151] Baron MS, Vitek JL, Bakay RA (2000). Treatment of advanced Parkinson's disease by unilateral posterior GPi pallidotomy: 4-year results of a pilot study. *Movement Disorders*.

[152] Obeso JA, Olanow CW, Rodriguez-Oroz MC, Krack P, Kumar R, Lang AE (2001). Deep-brain stimulation of the subthalamic nucleus or the pars interna of the globus pallidus in Parkinson’s disease. *The New England Journal of Medicine*.

[153] Rodriguez-Oroz MC, Obeso JA, Lang AE (2005). Bilateral deep brain stimulation in Parkinson’s disease: a multicentre study with 4 years follow-up. *Brain*.

[154] Volkmann J, Allert N, Voges J, Sturm V, Schnitzler A, Freund HJ (2004). Long-term results of bilateral pallidal stimulation in Parkinson’s disease. *Annals of Neurology*.

[155] Moro E, Lozano AM, Pollak P (2010). Long-term results of a multicenter study on subthalamic and pallidal stimulation in Parkinson’s disease. *Movement Disorders*.

[156] Alvarez L, Macias R, Pavón N (2009). Therapeutic efficacy of unilateral subthalamotomy in Parkinson’s disease: results in 89 patients followed for up to 36 months. *Journal of Neurology, Neurosurgery and Psychiatry*.

[157] Krack P, Pollak P, Limousin P, Benazzouz A, Deuschl G, Benabid AL (1999). From off-period dystonia to peak-dose chorea. The clinical spectrum of varying subthalamic nucleus activity. *Brain*.

[158] Stolze H, Klebe S, Poepping M (2001). Effects of bilateral subthalamic nucleus stimulation on parkinsonian gait. *Neurology*.

[159] Simuni T, Jaggi JL, Mulholland H (2002). Bilateral stimulation of the subthalamic nucleus in patients with Parkinson disease: a study of efficacy and safety. *Journal of Neurosurgery*.

[160] Krack P, Batir A, Van Blercom N (2003). Five-year follow-up of bilateral stimulation of the subthalamic nucleus in advanced Parkinson's disease. *The New England Journal of Medicine*.

[161] Rodriguez-Oroz MC, Zamerbide I, Gyridi J, Palmero MR, Obeso JA (2004). Efficacy of deep brain stimulation of the subthalamic nucleus in Parkinson’s disease 4 years after surgery: double blind and label evaluation. *Journal of Neurology, Neurosurgery and Psychiatry*.

[162] Deuschl G, Schade-Brittinger C, Krack P (2006). A randomized trial of deep-brain stimulation for Parkinson's disease. *The New England Journal of Medicine*.

[163] Hamani C, Richter E, Schwalb JM, Lozano AM (2005). Bilateral subthalamic nucleus stimulation for Parkinson’s disease: a systematic review of the clinical literature. *Neurosurgery*.

[164] Follett KA (2004). Comparison of pallidal and subthalamic deep brain stimulation for the treatment of levodopa-induced dyskinesias. *Neurosurg Focus*.

[165] Limousin P, Krack P, Pollak P (1998). Electrical stimulation of the subthalamic nucleus in advanced Parkinsonian’s disease. *The New England Journal of Medicine*.

[166] Montgomery EB, Baker KB (2000). Mechanisms of deep brain stimulation and future technical developments. *Neurological Research*.

[167] Vingerhoets FJG, Villemure JG, Temperli P, Pollo C, Pralong E, Ghika J (2002). Subthalamic DBS replaces levodopa in Parkinson’s disease: two-year follow-up. *Neurology*.

[168] Russmann H, Ghika J, Villemure JG (2004). Subthalamic nucleus deep brain stimulation in Parkinson disease patients over age 70 years. *Neurology*.

[169] Molinuevo JL, Valldeoriola F, Tolosa E (2000). Levodopa withdrawal after bilateral subthalamic nucleus stimulation in advanced Parkinson disease. *Archives of Neurology*.

[170] Figueiras-Mendez R, Marin-Zarza F, Molina JA (1999). Subthalamic nucleus stimulation improves directly levodopa induced dyskinesias in Parkinson’s disease. *Journal of Neurology Neurosurgery and Psychiatry*.

[171] Rodriguez-Oroz MC, Gorospe A, Guridi J (2000). Bilateral deep brain stimulation of the subthalamic nucleus in Parkinson’s disease. *Neurology*.

[172] Obeso JA, Rodriguez-Oroz M, Marin C (2004). The origin of motor fluctuations in Parkinson’s disease: importance of dopaminergic innervation and basal ganglia circuits. *Neurology*.

[173] Saint-Cyr JA, Hoque T, Pereira LCM (2002). Localization of clinically effective stimulating electrodes in the human subthalamic nucleus on magnetic resonance imaging. *Journal of Neurosurgery*.

[174] Voges J, Volkmann J, Allert N (2002). Bilateral high-frequency stimulation in the subthalamic nucleus for the treatment of Parkinson disease: correlation of therapeutic effect with anatomical electrode position. *Journal of Neurosurgery*.

[175] Hamel W, Fietzek U, Morsnowski A (2003). Deep brain stimulation of the subthalamic nucleus in Parkinson’s disease: evaluation of active electrode contacts. *Journal of Neurology Neurosurgery and Psychiatry*.

[176] Guridi J, Obeso JA, Rodriguez-Oroz MC, Lozano AA, Manrique M (2008). L-dopa-induced dyskinesia and stereotactic surgery for Parkinson’s disease. *Neurosurgery*.

